# How to Exercise During Coronavirus Quarantine?

**DOI:** 10.22088/cjim.11.0.479

**Published:** 2020

**Authors:** Rastegar Hoseini

**Affiliations:** 1 Department of Exercise Physiology, Faculty of Sport Sciences, Razi University, Kermanshah, Iran

**Keywords:** Coronavirus, Exercise, Infection

## Abstract

The COVID-19 pandemic caused stress and anxiety in many people that can be reduced by regular physical activity. Regular physical exercise is essential for health. In the absence of COVID-19 symptoms, no limitation in physical activity is recommended. However, parameters such as frequency, intensity, type, and time need to be considered to prescribe the program and obtain the best results. Consequently, the level of physical activity that should be done during the outbreak has always been one of the most important and common questions.

The Coronavirus disease (COVID-19) first spread in China. It has led to 962 thousand deaths and at least 31.1 million infections worldwide from December 2019 to 22 September 2020 (1). Germany, Italy, France, Canada, the United States, as well as several Asian countries, have reported a high incidence ever since. Iran's government reported 425000 people infected, and 24478 died. COVID-19 infects a new person through their mouth, nose, or eyes after exposure to air or a surface contaminated by sneezing or coughing of a patient (2). Governments have closed all sports and exercising centers to keep people safe from the coronavirus (3). Although quarantine and socio-physical distancing effectively decelerate the spread of the virus, they may have negative psychological and physiological effects on most people in the community that lead to nonconformity of health protocols (4, 5). Regular exercise and physical activity improve physical fitness, mental health (reduces depression, anxiety, and aggression) (6), reduces the incidence of chronic diseases (7), physical disabilities (8), and enhances the immune system (8). The ACSM has also recently identified physical activity as an effective factor in combating the complications and mortality of COVID-19 (4, 9). However, studies show a decrease in the individual’s physical activity level during the COVID-19 epidemic (10). Due to the positive physiological-psychological effects, performing physical activity seems necessary in the quarantine in all uninfected individuals (11, 12). However, these activities must be performed, considering a safe FITT (frequency, intensity, type, and time). 

## Methods

The present study reviews the published studies from 2000 to 2020 on the immune system and published studies from 2019 to 2020 on COVID-19. To find the relevant articles, the keywords coronavirus, exercise, and immune system were searched in Google Scholar, PubMed, and Science Direct sites.

## Results

For all individuals of any age, being physically active is essential to be healthy. For this purpose,150-300 minutes of aerobic training with moderate-intensity and two sessions of resistance training is recommended per week (13).

**Frequency: **Generally, 3 days per week for beginners and 3-5 days per week are recommended in this quarantine situation for athletes, regarding that volume and intensity must be adapted. The higher the intensity, the lower the frequency (10, 14).

**Intensity: **Exercise can be classified as low, moderate, and high based on its intensity. The exercise intensity is determined by evaluating blood lactate levels, the maximum oxygen consumption (VO_2max_), and the maximal heart rate (HR_max_). In low or moderate intensities, the lactate production remains steady between 2 and 4 mmol/L) (15). While studies commonly use a percentage of VO2_max_ and HR_max_ to express intensity. Thus, 20 to 50% of the VO_2max_ and the HR_max _refers to mild, 50–70% shows moderate, and above 80% is known as intense (16, 17). The acute effects of a moderate exercise session on immunology cells are well established (18-20). Different types and intensity may affect the immunological system diversely. While intense exercise weakens the body’s defense mechanisms, moderate-intensity exercise seems to improve them (20, 21). Intense exercises lead to neutrophilia, lymphopenia, and monocytosis (22). In comparison, moderate exercise mediates the redistribution of these cells in the vascular compartment by creating catecholamine’s (mostly adrenaline) dose-response in individuals (23). The b–receptor’s expression in the different immune cells (24), the adrenergic receptor’s density, and the efficiency of the AMPc transduction system may provide the molecular grounding for the action of lymphocyte and many other substances (25, 26). By a decreasing order, the neutrophils, NK cells, TCD8+ lymphocytes, the B lymphocytes, and finally, the TCD4+ lymphocytes seem to present a more significant number of receptors (27, 28). Intense exercise may induce the higher activity of the NK cells, the proliferative lymphocytic response, and the plasmacytic production of antibodies (29, 30). These changes may weaken the immune system against infection, oncogenic agents, allergic processes, and autoimmunity (31, 32).

On the other hand, increased leukocytes function had been observed after moderate-intensity interventions. Many researchers verified improvements in the oxidative activity of the neutrophils, chemotaxis, phagocytosis, and degranulation one hour after a moderate-intensity (60% VO_2max_) exercise (33, 34). Therefore, establishing a link between moderate-intensity exercises and promoting the immunological system is plausible. It is generally assumed that intense training protocols (>75% of O2max) and exhaustive competitions higher the risks to acquire upper respiratory tract infection (URTI) (35). Exaggerated production of ORS and an increase of oxidative stress in the tissues are the primary mechanisms by which high-intensity exercises suppress the immune system (36, 37). Changes in the body temperature, cytokine concentrations, stress hormone level, dehydration, and increased blood flow are the likely mechanisms involved in immune system responses to moderate-intensity exercise (38, 39). Overall, moderate-intensity predominates the Th1 cells in the immune response, thus promotes protection against infections (40). Conversely, high-intensity prevails the Th2 pattern responses to decrease damage in muscular tissue, causing an increase in susceptibility to infections (41, 42).

**Type of exercise: **Compared to a sedentary lifestyle, being regularly active is known to boost the immune system (43). The primary mechanism of aerobic exercises might be through changing antibodies and white blood cell levels in circulation and causing a brief rise in body temperature during and right after exercise that may help the body fight infection better similar to what happens in pyrexia. However, resistance exercise can reduce the chance of infection indirectly by slowing down the stress hormone secretion. Generally, some studies reported that aerobic training stimulates a pro-inflammatory response and decreases the risk of infection. Others noted that it might promote a decrease in these same parameters, increasing the risk of infectious diseases (44, 45). The same contradictory results are present for resistance training, as well (43). It seems that the differences in intensity may lead to conflicting results; Thus, the intensity is known to affect the immune cell responses rather than resistance or aerobic training (16, 17).

**Time: **Prolonged vigorous exercise elevates the serum cortisol level above the normal values (46). The suppression of both NK and T-cell function and production during recovery after a prolonged exercise is known to be related to Glucocorticoid levels (47). It causes lymphocytopenia, eosinopenia, and neutrophilia as well (38, 39). The neutrophil/lymphocyte ratio, which is the indicator of systemic inflammation, rises sharply after heavy, prolonged exercise (48). Generally, these shreds of evidence convey that prolonged endurance activities are associated with decreased host protection, immunosuppression and higher infection risk (49). It is also believed that stressful physical exercise may lead to similar outcomes (50). However, few convincing data are supporting the theory that elite endurance athletes have higher risks of infection. Further research is needed.

## Discussion

**Prescribing an Applicable and Practical Exercise program: **Nevertheless, moderate (<60% VO2max, <60 minutes/bout) versus vigorous (>75% VO2max, >90 minutes) exercise reduces the stress hormone response and enhances the immune defense. Considering the convincing evidence, advising low to moderate intensity exercise with shorter duration is more prudential. The following is recommended accordingly;

**Indoor Activities**

- Walk briskly with music on around the house or climb the stairs for a total of 30-45 min per day (10- 15 min, 2-3 sets)
- Dance to a favorite music total of 15 min
- Butterfly, scissors, squat, step-ups, planks, and jumping without a rope (if having healthy joints)
- Participate in an online exercise workout
- Exercise with a treadmill, rowing machine, spinning bike, step mill (If you have them at home)

**Strength Training**

- Use strength workouts that need no equipment
- Inspire from bodyweight training videos
- Increase the resting period
- Perform yoga with deep breathing
- Perform meditation and mindfulness

**Outdoor Activities (if allowed by your government): **However, outdoor activities are not recommended, keep staying 6 feet away from others while doing outdoor activities, not touching the face, and washing the hands and clothes when geting home:

- Walking or jogging around
- Spending time in nature may enhance immune function.
- Riding bicycle in solitude
- Tending the lawn and garden
- Play active games with family

A graphical abstract is also included below:

**Figure 1:**
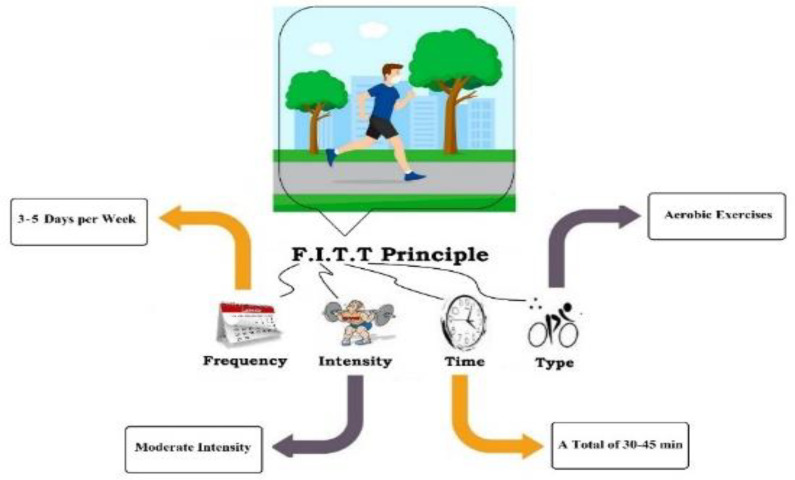
Contents Graphic (Graphical Abstract)

## Conflict of interests:

The authors declare that they have no competing interests.
